# A new approach to study the relative age effect with the use of additive logistic regression models: A case of study of FIFA football tournaments (1908-2012)

**DOI:** 10.1371/journal.pone.0219757

**Published:** 2019-07-16

**Authors:** Miguel Saavedra-García, Marcos Matabuena, Antonio Montero-Seoane, Juan J Fernández-Romero

**Affiliations:** 1 Grupo de Investigación en Ciencias del Deporte (INCIDE). Departamento de Educación Física y Deportiva, Universidade da Coruña, A Coruña, Spain; 2 Centro Singular de Investigación en Tecnoloxías da Información (CiTIUS), Universidade de Santiago de Compostela, Santiago de Compostela, Spain; Instituto Politecnico de Viana do Castelo, PORTUGAL

## Abstract

The relative age effect plays an important role in the pursuit of excellence, providing advantage to athletes born at the beginning of the year or near the cut-off date. This phenomenon has been observed in areas such as sports, education or business. Traditionally, the chi-square test has been used to analyze whether there are statistically significant differences in the distribution of births in each of the four quarters of the year. However, this approach is limited, focusing only on the analysis of the response variable, without taking into account the effect of a set of predictive variables. In this paper a new approach is proposed to study the relative age effect with the use of a logistic regression additive model. The new method has been evaluated with a sample of 21,639 players involved in football tournaments organized by the Fédération Internationale de Football Association (FIFA) between 1908 and 2012. New conclusions have been established that the relative age effect exists regarding player age and the year of the competition in male FIFA competitions and its effect is dynamic and complex.

## Introduction

The establishment of factors associated with sports excellence in football has been a very popular topic in the physical exercise science literature. Traditionally, attention has only been paid to the physiological [1–3], biomechanical [4, 5], technical and tactical [6, 7] characteristics of professional football players. Some variables that have been taken into account for an exhaustive analysis are maximum oxygen consumption [8], levels of force of afferent muscles [9], acceleration capacity from a 30 m sprint, and other variables that measure the motor skills of athletes during game performance [10] and while performing certain technical tasks [6] and the ability of players to concentrate [11].

However, there are other variables not linked directly to player performance in a match, such as date of birth [12, 13], family environment during adolescence [14], or birthplace and country of residence [15, 16], which for sociological reasons increase the chances of a young man becoming a professional athlete.

The relative age effect (RAE) is a concept that refers to subjects having dates of birth near the beginning of the year or near the cut-off date. These athletes are more likely to achieve excellence in a field over those who are born later. This phenomenon has been observed in several sports, such as basketball [12, 13], rugby [10], ice hockey [15], tennis [17], athletics [18] or football [13]. Additionally, the RAE has had a great impact in other areas such as education, i.e., obtaining better grades [19, 20], teacher perceptions of pupil behavior [21], political selection [22] and youth suicide [23].

The main explanation of the existence of the RAE is that the subjects born at the beginning of the year, or closer to the cut-off date, when they are children, tend to have advanced physical and mental development [14]. This is very important in football because physical aspects are more important than technical skills at those ages [24]. In this way, children born in the first part of the year have development advantages, and they are considered better players. Consequently, they play more hours in official competitions, and as a result, the extent of their abilities increases.

A strong RAE was detected in footballers between 6 and 18 years old in higher and intermediate expertise teams, but not in low expertise teams [25]. In Australian footballers, the RAE was documented in ages between 10–12 years and continued into senior professional competition [26]. The same observation was found in Spain, where the RAE has been increasing during recent years in youth elite competition [27], and continues into elite sports [28]. The RAE was evident in professional male football players of the ten best leagues of Union des Associations Européennes de Football (UEFA) [29], and is well recognized in female football as well [30, 31].

The classical statistical procedure for the study of the RAE is to test for statistically significant differences between the number of athletes born in any of the four quarters of the year based on the use of a chi-square test to compare an expected uniform distribution [12, 13, 17, 18, 20, 25, 26, 28, 29, 32, 33] or a nonuniform excepted distribution [17, 18, 34]. In some cases, for example, when the study sample is small, Fisher’s exact test is used [17].

However, these approaches do not allow the exploration of how the characteristics of athletes affect the RAE, focusing only on separately analyzing the distribution of the number of births per quarter. Furthermore, in the literature, there are several questionable methodological practices in the statistical procedures [35]; therefore, the results shown in the articles may not be entirely correct. Frequently, several case studies are analyzed, and therefore, several hypotheses are simultaneously tested. In this situation, from a statistical point of view, a correction procedure must be applied for multiple comparisons [36], something that is not carried out consistently in the literature and that causes, in practice, p-values to be more optimistic than they really are. Moreover, many of the observations in the studies may not be independent due to bias caused by sampling. Given the observational nature of some of these studies, the presence of latent variables, such as anthropometric characteristics of the athlete [37], or other demographic, legislative (changes in the regulations that classify athletes in one category or another) and economic [38] variables in some subsets of a sample or between studies prevents an effective comparison between studies without first fixing the sources of variation for these variables. One way to overcome those limitations is to use a predictive model that has as covariates product of these variations or biased results. At the same time, this approach allows analysis of the effect of each of the predictor variables on the effect of relative age, with the possibility of verifying whether the effect obtained is statistically significant.

In recent years, new articles have been published that analyze the RAE using regression models. For example, in [39], using a quantile regression on salaries, an inverse effect of the RAE was observed. In [15, 40, 41], Poisson’s linear regression models were used to analyze the number of births, and in [42] a negative binomial distribution was employed. However, few studies exploit the possibility of analyzing a set of athlete variables [40, 41] and, instead, focus on analyzing characteristics of the athlete individually. Additionally, it is possible that the RAE tends to provide a greater overrepresentation of subjects born at the beginning of the year or near the cut-off date. It is likely not necessary to quantify how the number of athletes born evolves during all years, as done in [15, 43, 44]; in practice, it is more interesting to analyze a categorized variable that divides the year into different periods. In this way, the variability in the model is reduced, while the interpretability of the model increases, with the advantage that in this case, the objective is to estimate the probability of each class and the effects of each variable and non-absolute value (which depends on the size of the database). The adjustment of these models can be done, for example, with logistic regression [20, 45] or, in the case of more than two classes, with multinomial regression [46, 47].

Generalized additive regression models are predictive models that allow modeling nonlinear relationships between a set of predictors and a variable response. Their use is widespread in many scientific fields, such as ecology [48] or epidemiology [49], because they are highly interpretable models that allow quantifying the effect of each value of each explanation on the response, addressing one of the limitations of linear multivariate regression models that assumes an effect proportional to the value of the adjusted coefficient.

Although in the case of football [33, 50, 51] and other sports [12, 15] many studies have observed the existence of the RAE, it is still necessary to study this issue more deeply and contemplate new aspects such as the influence of economic, sociological, psychological and biological variables on the RAE.

The aim of this paper is to establish a new methodology to study the RAE in the presence of several predictive variables through the use of an additive logistic model. For this, we will analyze a sample of all players in FIFA football competitions from 1908 to 2012, comprising 21,639 male football players. The considered variables included the year of the competition, age of the player in the championship and playing position on the field. An additive regression model was fitted using the variables defined previously with the variable responses birth in the first quarter of the year. In this paper we analyzed, for the first time, field issues such as the evolution of the RAE over time and the age of the athlete.

## Materials and methods

### Sample

Using the FIFA website (http://www.fifa.com/tournaments/archive/), information from more than 36,690 football players (31,022 men with age 22.60±5.03 years and 5,668 women with age 20.25±4.63 years) who played in tournaments organized by FIFA between 1908 and 2012 was collected.

Taking into consideration all the tournaments available, nine men’s (FIFA World Cup, FIFA U-20 World Cup, FIFA U-17 World Cup, FIFA Confederations Cup, Olympic Football Tournament, FIFA Club World Cup, FIFA Futsal World Cup, FIFA Beach Soccer World Cup and Youth Olympic Football Tournament) and five women’s (FIFA Women’s World Cup, Women’s Olympic Football Tournament, FIFA U-20 Women’s World Cup, FIFA U-17 Women’s World Cup and Youth Olympic), only the men’s football competitions were selected. The research protocol was approved by the ethics committee of the Universidade da Coruña (CE 21/2015).

### Variables

The variables obtained for all the football players were the championship, sex of the athlete, FIFA confederation, year of the competition, location of the competition, team, specific position on the field, and date of birth. Height was also available for a very low proportion of players; therefore, this variable was discarded for the analysis. Moreover, the specific position on the field was not available for more than 7,000 players; therefore, to not lose an important sample size in the statistical analysis, players without a position recorded were included.

Consequently, using the date of birth, the competition and the year in which the competition was held, five new variables were created:

- Quarter: Binary variable that indicates whether the player was born between January 1 and March 31;
- Player_age_Championship: Continuous variable that indicates the age of the player at the beginning of the championship.

### Statistical analysis

#### Procedure

Before starting, we removed repeated data: a single datum was chosen from each player chosen at random (introducing a seed equal to 25 in the statistical software R) to avoid invalidating the statistical inference analysis. Then with the variables Player_age_Championship, Year_Competition and Position_Player, an additive logistic regression model was adjusted (with 21,639 players) using the “Quarter” variable as response. The formula for the model introduced in the statistical software R with the gam function of the mgcv package is: model = gam(Quarter~Position_Player+s(Year_Competition)+s(Player_age_championship),family = binomial(),data = datos). The term *s ()* indicates that the variables were introduced in an additive way. For more information on the statistical procedure used and computational issues, see the following reference [52], in which the estimation of the model was carried out by penalized likelihood instead of the classic backfitting procedure. The graphics were created with the default plot function of the mgcv package.

A multiple testing correction procedure under dependence of the p-value was applied to all of the calculated p-values. The null hypothesis for each categorized variable is that the odds ratio is equal to 1 with respect to the reference category, while for the continuous variables, the null hypothesis that the effect between the predictor variable and the response is null.

Finally, we established a cut of significance equal to a p-value of 0.05.

#### Additive model

The main novelty of this work is the use of a generalized additive model. Its use allows, on the one hand, analyzing nonlinear relationships between the predictors and the response and, on the other, interpreting those relationships. An advantage of the method used in this study is that it enables an analysis of the effect of each year of competition, athlete age, and RAE, as opposed to what occurs with a multivariate linear regression or classical generalized model, which assigns an overall effect for each variable.

#### Interpretation of odds ratios and odds for the RAE

The key element for interpreting the effect of a covariable X on a response variable Y in a logistic regression is the odds ratio. An odds ratio is a statistical measure that quantifies the probability of an event occurring with respect to another reference. Given two possible values of the covariate X, x1 and x2 which indicate two different values for an athlete characteristic (e.g. position on the field or year of competition), the odds ratio of x2 to x1 is defined as follows:

oddsratio(x2vsx1)=(P(Y=1/X=x2)/P(Y=0/X=x2))/(P(Y=1/X=x1)/P(Y=0/X=x1)).

Note that in the above definition, x1 represents the reference value, and the odds ratio measures how much more likely it is that Y = 1 will occur if X = x2, with respect to X = x1. An odds ratio equal to 1 means that there is an equal probability of occurrence, while an odds ratio greater than 1 indicates that Y = 1 is more likely to occur if X = x2, and an odds ratio less than 1 indicates that Y = 1 is more likely if X = x1.

In the case of a logistic regression model, the odds ratio of variable X is equal to the exponential of the coefficient associated with that variable or of the so-called estimated value. When X is a categorical covariate, its value is interpreted used the reference category previously established in the analysis. For continuous variables, the value of the predictor variable increases in units of one (odds ratio ((x1 + 1) vsx1). The interpretation of the exponential of the intercept is different, in this case we calculate the odds instead of the odds ratio, that is, P (Y = 1) / P (Y = 0) with respect to the reference categories of the variables and without the effects of continuous variables.

In this paper, the odds of the intercept indicates the global effect of relative age when there is no effect of continuous covariates and categorical variables take the reference values. However, because we are studying the proportion of births of the first 90 days of the year, in relation to the remaining 275 days, we have to calculate the reference values for the odds in the context of a uniform distribution, that is, (90 * 365) / (275 * 365) = 0.327. Therefore, an odds in the intercept greater than 0.327 is indicative of an effect of relative age, whereas otherwise, it does not. Additionally, given the odds, we can estimate the number of days that this value would correspond in the context of a uniform distribution using the formula (365 * odds) / (1 + odds), which can be seen as a measure of the strength of the relative age effect.

## Results

The additive regression model allows calculating the estimation value for each value that the continuous explanatory variables can take.

**Fig 1:**
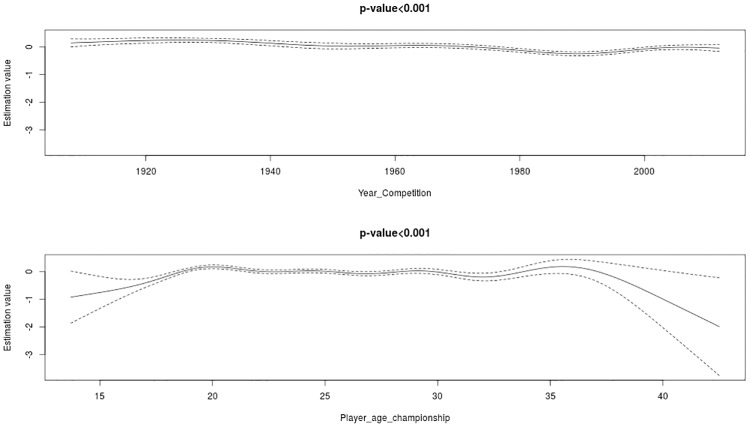
Effects of the variables introduced in an additive manner into the model.

In Fig 1, the effects of the variables introduced in an additive manner into the model are shown, together with their p-values adjusted for each of the study response variables. In parallel, Table 1 shows the odds ratios, raw confidence intervals (CIs), and adjusted p-values for the categorical variables and intercept.

**Table 1:** Results categorical variables and intercept in additive logistic regression model.

	Odds/Odds ratio	Raw confidence interval (95%)	Adjusted p-value
(Intercept)	0.439	0.408–0.473	<0.001
Position_Player: No_specified	0.980	0.885–1.087	1
Position_Player: Defense	0.999	0.905–1.105	1
Position_Player: Forward	0.919	0.829–1.017	0.388
Position_Player: Goalkeeper	0,888	0.784–1.005	0.337

Reference category: Midfield

The two continuous variables are statistically significant with a p value <0.001. In the variable Year Competition the relative age effect is not constant over time; there are small oscillations around the estimation value of 0; however it is quite stable over time. In addition, a small increase in the estimated value in the last 15 years is observed. Regarding the variable Player_age_Championship at younger ages, younger athletes see a lower RAE that increases until 20 years of age, when it stabilizes until approximately 35 years of age. From that age, the RAE decreases again, in a significant manner.

The categorical variable Position_Player shows no statistically significant differences with respect to the reference category. The smallest odds ratio is 0.888 (p-val = 0.337) for the goalkeeper position. The intercept yields a strong RAE with an odds ratio of 0.439 (under the context of uniformity, the reference value is (90 * 365) / (275 * 365) = 0.327; the obtained value is not within the confidence interval), which indicates that in the first 90 days of the year, as many athletes have been born as would be expected in 111.35 days.

## Discussion

The relative age effect is a topic widely studied in the literature in areas as diverse as education [19, 20], sports [12, 13] and youth suicide [23]. However, until now, there has not been a full consensus regarding its definition [53], referring generally to a greater overrepresentation in the number of births in the first three months of the year. Given this inaccuracy, this paper proposes a new methodological approach for the study of RAE. This approach consists of the use of an additive logistic regression model [54], which classified if a subject was born in the first x days of the year or, on the contrary, in the rest of the year.

In this work, the established methodology uses the first 90 days of the year, or a quarter, which is the time margin that is usually used when quantifying the RAE. The purpose for choosing this timespan is to be consistent with the published literature and, in this way, create a corpus of common knowledge. However, in future studies, it would perhaps be more appropriate to establish an optimal cut-off point derived using a data-driven approach. However, for this, it is necessary to develop specific strategies to categorize the response variables into two groups based on the predictive capacity of the model and its significance. To the best of our knowledge, there are only statistical tools that automatically categorize predictor variables [55], not response variable.

In other works, the number of births on each day of the year was estimated with Poisson regression [40, 56, 57]. However, this approach, in practice, does not help to quantify in a direct way an overrepresentation of the number of births in a determinate period; because, this method has inconvenient estimates of absolute quantities, which hampers the effective comparison between databases of different sizes. It is true that these absolute quantities can be transformed into proportions; hence, there is proximity between Poisson regression and logistic regression for this problem, but the interpretation of the regression coefficients for each model and the inferential procedures are different.

Some authors have attempted to quantify the RAE with Poisson linear regression using an index of discrimination and an index of wastage respectively [57]. Both rates are proportions, and, for example, the former was used to measure the ratio of the number of births expected at the beginning of the year to the end of the year. However, these indexes are only valid if the underlying model is correctly specified and there are monotonic relationships in the number of births. As another disadvantage, they can only be applied if the regression model is univariate. In this work, on the contrary, odds have been determined as possible indicators of the strength of the relative age effect together with the expected number of days in a context of independence with those odds. This approach is valid with multivariate regression models; however, the validity depends on the previously chosen reference categories, and the model should be interpreted with the possible effect of continuous variables. Other advantages of the proposed indicators are that hypotheses can be directly contrasted with the confidence interval of the odds, unlike an index of discrimination, for which the derivation of the statistic and the distribution of the contrast is more laborious and often times not determined. Additionally, with the understanding that the RAE indicates a greater overrepresentation at the beginning of the year regarding the number of births, it may not make sense to use what happens at the end of the year as a reference element in the measurement of the RAE, as the reference index does, it make more sense to compare only the first x days of the year against the rest of the year as the odds do.

**Fig 2:**
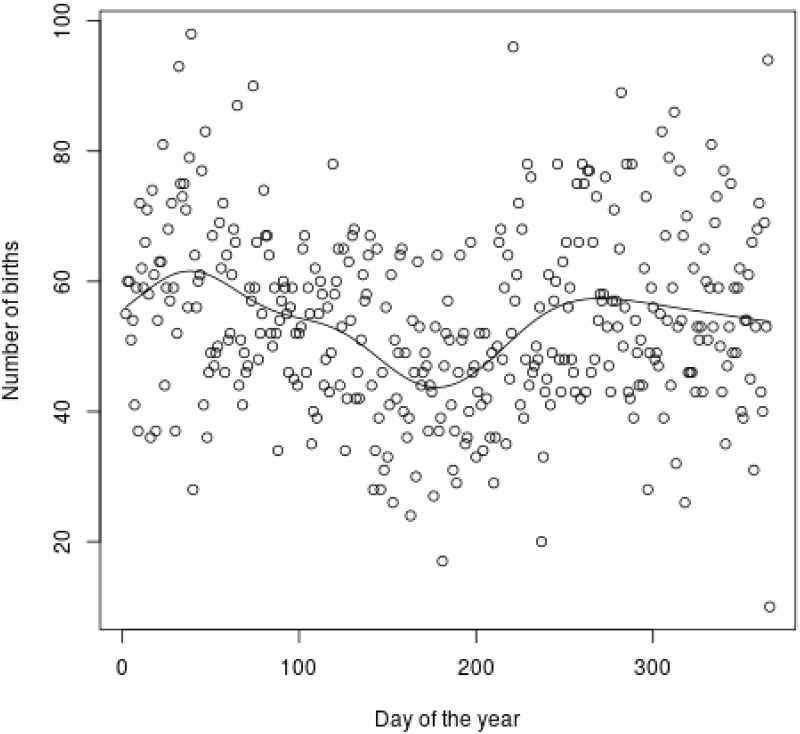
Births in each day of the year.

Additionally the Poisson regression methods used were linear [40, 57], which seems to be a strong restriction in practice because the number of births and its relation to the covariates does not have to be linear, having in many situations an overrepresentation of births in the first and third quarters of the year or in clustered time points within the year, e.g., around special holidays. To better illustrate this fact, Fig 2 shows the number of births each day of the year together with the nonparametric estimation of a Poisson regression model, supporting the aforementioned claim.

In this paper, this last limitation is addressed using an additive model that is able to model nonlinear relations of association between the predictor variables and the variable response. In addition, these models are highly interpretable and allow analysis of, for the first time, the effect of relative age on the year of the competition, the age of the athlete, and his or her height, providing interesting results.

The RAE is not constant over time, having a greater effect at the beginning of FIFA football competitions and in competitions of this century; however there are no substantial variations. Likewise, in younger players with a lower professional component, there is a lower RAE that increases progressively and quite remarkably until 20 years of age when it stabilizes until 35 years of age and then decreases.

Regarding the position on the field, no statistically significant differences were found, which indicates that this variable is not a true element that differentiates the RAE. Other variables, such as year of competition, or age of the player, are most likely more important.

The value of the odds of the intercept yields a strong overall RAE in this database. In quantitative terms, in the first 90 days of the year there are as many births, as would be expected in 111.3 days, which indicates that the RAE exists and is important. This effect is remarkable, especially in players between 20 and 35 years of age when the estimated value is close to zero. Furthermore, the year of competition does not oscillate significantly away from the estimated value of zero; this effect remains stable over the long term.

These results support the idea that the phenomenon of the RAE exists but that its etiology can be dynamic and multifactorial in nature. In this sense, more research that utilizes prediction models to exploit the possibility of analyzing a set of covariates is required. Some examples include quantifying the athletic level of the athlete and, the degree of biological maturation at younger ages (where it is observed that the RAE is lower) and, using anthropometric variables or other geographical and socioeconomic variables.

One of the limitations of the present study is that the variables introduced only provide a partial explanation of the RAE that is, the effect over time considering the age of the player. However, given the observational nature of most relative age studies, it would be interesting to take into account the variables discussed above to delve more deeply into the mechanisms that produce the RAE. Another limitation is that not all countries have the same cutoff point on January 1, as is the case in England, where the RAE may be observed at another time of the year. In spite of this, we have detected an important effect of relative age that could be greater by further controlling biases.

## Conclusions and practical applications

In this work, a new methodology to study the RAE with the use of additive logistic regression models has been proposed. The new method allows analysis of the RAE in the presence of covariates and model nonlinear relations between variables.

Lastly, this methodology was illustrated with an analysis of FIFA competitions and has demonstrated the effect of the RAE with age of the athlete, and year of the competition. The results show that the RAE exists and that, its effect is dynamic and complex.

In addition, there is already a known need to create competition calendars that avoid faults in the equality of opportunities between football players in competitions with a high degree of professionalism.

These results may help trainers and coaches. The efficacy of early talent identification based only on physical factors to predict future performance in elite sports is not the best method. Young players born later in the year and who can reach high skill levels in sports usually drop out because they feel less competent and have fewer opportunities.

## Supporting information

S1 File FIFA.sav. SPSS data for this study.
(SAV)
